# 3D Imaging Based on Depth Measurement Technologies

**DOI:** 10.3390/s18113711

**Published:** 2018-10-31

**Authors:** Ni Chen, Chao Zuo, Edmund Y. Lam, Byoungho Lee

**Affiliations:** 1 Department of Electrical and Computer Engineering, Seoul National University, Gwanak-Gu Gwanakro 1, Seoul 08826, Korea; chenni@snu.ac.kr; 2 Jiangsu Key Laboratory of Spectral Imaging & Intelligent Sense, Nanjing University of Science and Technology, Nanjing 210094, China; zuochao@njust.edu.cn; 3 Department of Electrical and Electronic Engineering, The University of Hong Kong, Pokfulam, Hong Kong, China; elam@eee.hku.hk

**Keywords:** three-dimensional imaging, computational imaging, light field, holography, phase imaging

## Abstract

Three-dimensional (3D) imaging has attracted more and more interest because of its widespread applications, especially in information and life science. These techniques can be broadly divided into two types: ray-based and wavefront-based 3D imaging. Issues such as imaging quality and system complexity of these techniques limit the applications significantly, and therefore many investigations have focused on 3D imaging from depth measurements. This paper presents an overview of 3D imaging from depth measurements, and provides a summary of the connection between the ray-based and wavefront-based 3D imaging techniques.

## 1. Introduction

**Figure 1:**
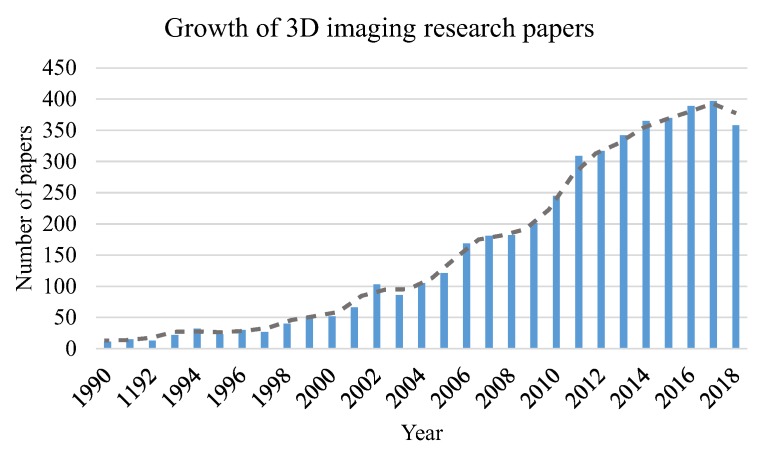
Number of papers with a title containing “3D imaging” in Google Scholar during the past decades.

Beyond the conventional two-dimensional (2D) imaging and photography, 3D imaging, which carries more information from the world, has a wide range of applications, particularly in the fields of life science [1] and information science [2]. Therefore, it has been attracting more and more attention in recent years [3,4]. Due to the large amount of various terminology definitions, it is difficult to show the survey about the depth measurement based 3D imaging techniques. Instead, we show in Figure 1 the growth of papers with “3D imaging” in their titles during the past decades searched for from https://scholar.google.com, which indicates increasing interest in 3D imaging to a certain degree.

**Figure 2:**
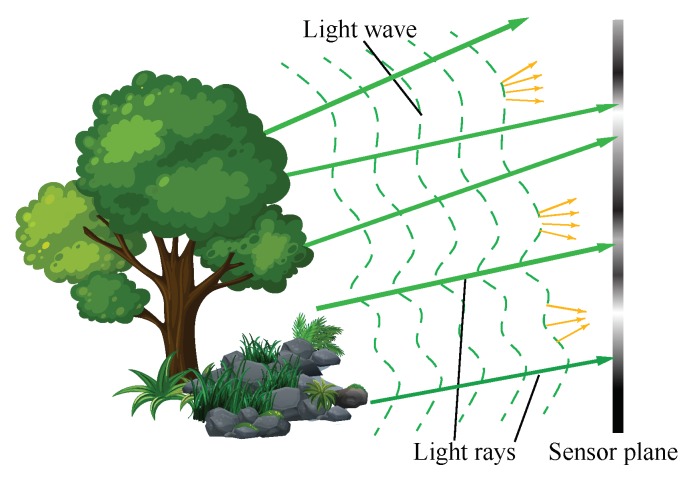
Relationship between light ray and wave.

When we refer to 3D information, we usually mean that, in addition to the 2D images, the depth or shape of the objects is also captured. The 3D information of an object is contained in the wave’s intensity and phase of the light wave over it. 3D imaging, in some cases, can be regarded as both amplitude and phase imaging of the object wavefront, which is also known as wavefront imaging. The phase of an electromagnetic wave is invisible, but it is inevitably changed as it is reflected by or passes through an object, which induces intensity changes. The reflection case is illustrated in Figure 2. The surface shape of the object bends or distorts the light that is reflected. In a transmission case, a phase delay of the wavefront due to the refractive index of object contains the information about the shape and density of the object. The amount of light bent behaves as a phase delay of the wavefront, and reflects the surface topology [5] or the shape and density [6] of the object. The detected intensity image of the wavefront thus carries the 3D information of the object, resulting in specific intensity patterns. The brighter areas reflect light energy concentration and dimmer areas reflect light energy spreading out, as shown in Figure 2. In the real world, objects may have absorption, which makes the measured intensity images affected by both refractive index and absorption.

As is commonly known, light waves and rays are used to describe light in different levels, while the Eikonal equation gives a link between ray optics and wave optics [7]. Wave optics is used to describe phenomena where diffraction and interference are important, while ray optics is where light can be considered to be a ray. In many imaging cases, treating light as rays is enough to solve the problems. From the viewpoint of ray optics, as shown in Figure 2, the directions of the reflected light rays are determined by the normal to the object surface at the incidence point [8]. Therefore, by recording the light rays coming from the objects with their directions, we can also reconstruct its 3D surface shape. Imaging with light rays along with its direction is called light field imaging [9,10]. In this paper, we name it as “light ray field” to distinguish from “light wavefront field”. Based on the above description, 3D imaging can be regarded as light ray and phase (wavefront) imaging to some extent.

**Figure 3:**
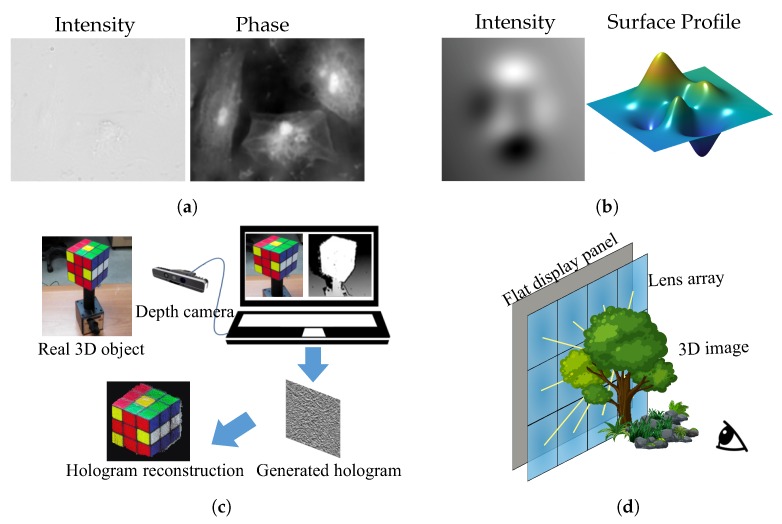
Applications of 3D imaging for (**a**) microscopy; (**b**) surface measurement; (**c**) holographic display; and (**d**) light field display.

The applications of 3D imaging are extremely wide. Figure 3 shows several of them with different 3D imaging techniques. Figure 3a shows an object’s structure with phase imaging, Figure 3b shows a surface profile of a wavefront, Figure 3c is an example of 3D imaging with holography, and Figure 3d is an example of 3D display based on light field 3D imaging. The wavefront-based 3D imaging is efficient for viewing structures in microscopy [11] and medical applications. The light ray field, which can produce multiple view images with parallax, is a practical and commercialized way for both stereoscopic and auto-stereoscopic 3D displays [12,13]. Benefiting from 3D imaging, hologram synthesis, which sometimes combines the wavefront-based and ray-based light field techniques, can make a hologram of the real world 3D scene under incoherent illumination, making holographic 3D display more realizable [14,15].

However, both “light ray” and “phase” of the wavefront cannot be detected directly. Based on the fact that intensity measurements carry the 3D information about the object wave, and benefiting from computational imaging [16], 3D imaging has become prosperous in recent decades [16]. Many techniques have been developed in phase imaging, including coherent diffraction imaging [17], phase retrieval [18,19,20], holography [21,22], time of flight (TOF) [23], and structured-light [24]. For light ray imaging, there are also a lot of techniques such as stereo imaging [25] and light field [9,26] (Stereo imaging can be regarded as an extreme light field imaging). Considering the imaging quality and the system complexity, recently, many investigations have been conducted on 3D imaging from depth measurements. In this paper, we present an overview of ray-based and wavefront-based 3D imaging using depth measurement techniques. Our paper is organized along a way from simple concept to in-depth description. Because ray optics gives us good intuition, we introduce ray-based techniques first, which is followed by the description based on wavefront-based techniques. For each type, we briefly introduce the theory and its technical development in Section 2. Then, we review the state-of-the-art depth measurement based techniques of the two types, respectively, in Section 3 and Section 4. Due to the close connection of the ray and wave optics, there is no clear boundary between the two types of 3D imaging techniques. In Section 5, we show comparisons between some of these techniques. Some concluding discussions are given in Section 6.

## 2. Theory of 3D Imaging and Progress of Its Development

In this section, we describe the fundamentals of ray-based and wavefront-based 3D imaging. It will reveal why 3D imaging with depth measurements becomes a trend.

### 2.1. Ray-Based Light Field Imaging

**Figure 4:**
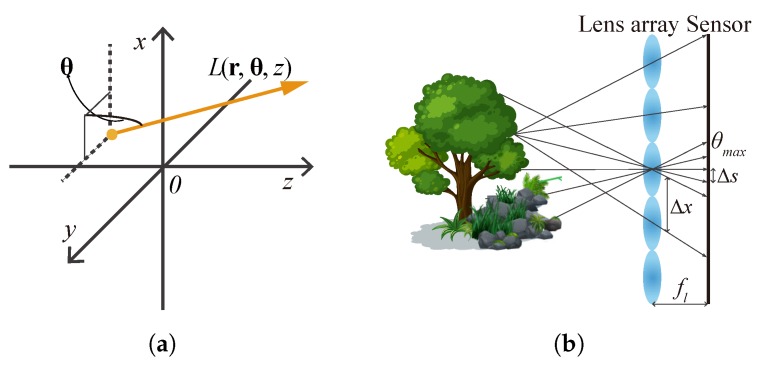
Light field (**a**) and its conventional capture (**b**).

As Figure 4a shows, according to the plenoptic function [27], light ray field can be parameterized as a function L(r,θ,z) with its spatial position (r,z) and propagation direction θ, where r is the position vector representing the transverse spatial coordinates (x,y) and θ is the propagation vector of $\left(\theta_{x},\theta_{y}\right)$. Because of the conventional capture approach, light ray field is usually expressed by two planes as a four-dimensional (4D) function [28]. In this paper, we use the former one for convenience. Assuming that the energy traveling along the optical rays is a constant, the 2D images detected by a camera at a plane perpendicular to the optical axis at $z_{c}$ can be expressed by angularly integrating the the light rays

(1) $$\begin{array}{c} I_{i}\left(r,z_{c}\right)=\frac{1}{z_{c}^{2}}\int_{-\infty }^{\infty }L\left(r,\theta ,z_{c}\right)d\theta . \end{array}$$

The light ray field can also be easily propagated from a plane at *z* to another plane at $z^{\prime }$ by [29]

(2) $$\begin{array}{c} L\left(r,\theta ,z^{\prime }\right)=L\left(\frac{r}{\alpha }+\theta \left(1-\frac{1}{\alpha }\right),\theta ,z\right), \end{array}$$

where $z^{\prime }=\alpha z$, and α is a real number. The transformation property of the light ray field makes it useful for 3D imaging such as depth map reconstruction [30] and digital refocusing [29,31]. In addition, it is also an efficient way for glass-free 3D displays [12,13,32].

Various light ray field acquisition methods have been developed, including complicated setups such as camera arrays [33,34], compact designs that utilize micro-lens arrays [29,35], frequency domain multiplexing [35], amplitude masks [36,37], and well-designed mirrors [38,39]. Among these techniques, a camera with a micro-lens array in front of its sensor [29,40,41] is well-known and widely used due to its single-shot convenience, as Figure 4b shows. In this kind of capture, every micro-lens captures angular distribution of the light rays at its principal point. The number of light rays that can be recorded depends on the lens pitch $\Delta_{x}$ and the pixel pitch $\Delta_{s}$ of the camera sensor. The maximum angle $\theta_{max}$ of the light rays that can be collected depends on the focal length $f_{l}$ and the lens pitch Δx. The spatial sampling interval of the object is the same as the pitch of the lens array. This lens array based method enables direct capture of the light field at a single shot, but the spatial resolution and angular resolution of the captured light field mutually restrict each other [12,13]. This mutual restriction occurs in the similar setups like camera array. During the past 15 years, many techniques have been proposed to enhance the spatial resolution, the view angle and the expressible depth range [13,42,43,44]. Unfortunately, there is no way to eliminate these shortages that were inherited from the array-like devices.

Based on the fact that depth measurements carry the 3D information of the objects, researchers have tended to retrieve a light ray field from depth measurements instead of using arrays. We review these techniques in Section 3.

### 2.2. Wavefront-Based Light Field Imaging

**Figure 5:**
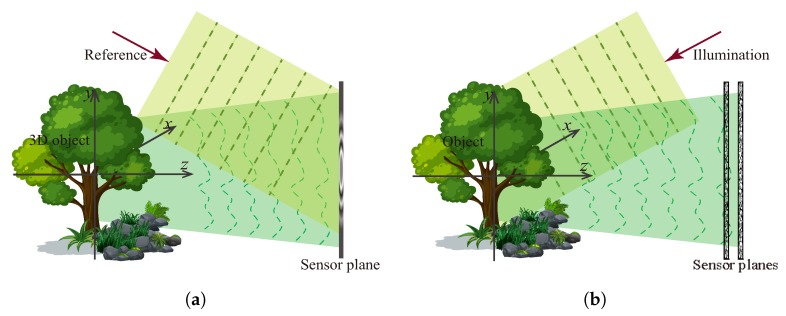
Two typical wavefront imaging techniques: interferometric (**a**) and phase imaging (**b**).

Wavefront-based light field imaging can mainly be categorized into two types: the interferometric approach and phase retrieval. The early wavefront light field imaging is based on the interferometry of waves, generally known as holography, which was first invented for microscopic imaging by Dennis Gabor [21] in 1948. As Figure 5a shows, the interference between the object wave and a known reference wave converts the object wave phase into intensity modulation, which makes it possible to reconstruct the phase both optically [45,46] and digitally [22,47].

However, introducing a reference beam requires a complicated interference experimental setup, which limits its applications and also induces some other problems in hologram reconstruction, such as the direct current (DC) term, twin-image, and speckle noise. Solving these problems usually requires a more complicated experiment setup [46,48,49]. Therefore, interferometric techniques are not well suited to imaging, such as an optical microscope that requires partially coherent light. It is necessary to remove the complexity and the coherent limitations of the interferometric techniques. Alternatively, this can be achieved by using out-of-focus images. We can use one intensity image measured with a known complex transfer function or a set of out-of-focus intensity images to estimate the phase quantitatively. This is known as phase retrieval. It takes the advantage of the development of computational imaging [16], leading to a versatile and simple experimental imaging system. Even optical components like lenses are non-essential in a phase imaging setup with depth measurements, as Figure 5b shows, which leads to a broader applications of phase imaging, such as X-ray imaging [50].

Phase retrieval usually can be achieved by iterative and quantitative approaches. Both only require one or a few depth measurements of the diffracted wave. In Section 4, we will review this kind of techniques.

## 3. Ray-Based Light Field Imaging from Depth Measurements

In light ray field reconstruction, the depth measurements are usually detected by a conventional camera such as a digital single-lens reflex (DSLR), under white light source. The formation of the photographic images has a close connection to the light ray field under geometric optics, and we will show this in the following paragraphs. After this, we will review the related techniques.

### 3.1. Focal Sweeping Measurement with a Conventional Camera

**Figure 6:**
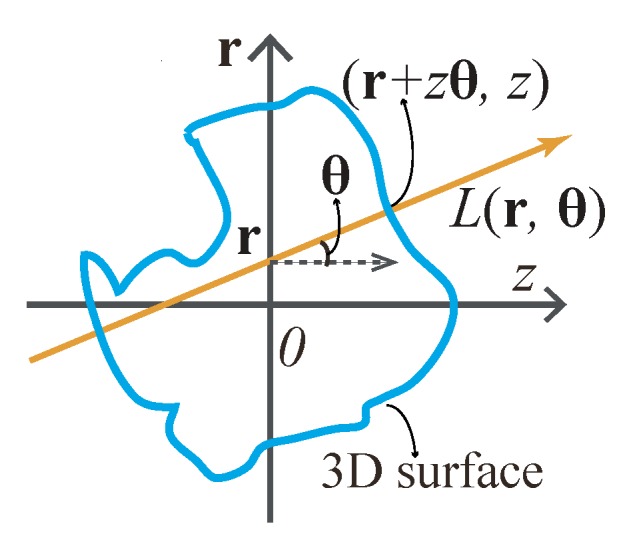
A 3D object’s light field representation.

We start from considering a 3D object with its center located at the origin of the Cartesian coordinates. Suppose the 3D object is regarded as a stack of 2D slices, i.e., O(r,z)=∫O(r;z). The light ray field representation of the 3D object with the principal plane located at its center can be expressed as the integral of the object’s projections by Figure 6 with [51]

(3) $$\begin{array}{c} L(r,\theta ,0)=\int O(r+z\theta ,z)dz. \end{array}$$

Figure 6 shows the geometry relationship between the 3D object surface and its light ray field on the r–*z* sectional plane.

**Figure 7:**
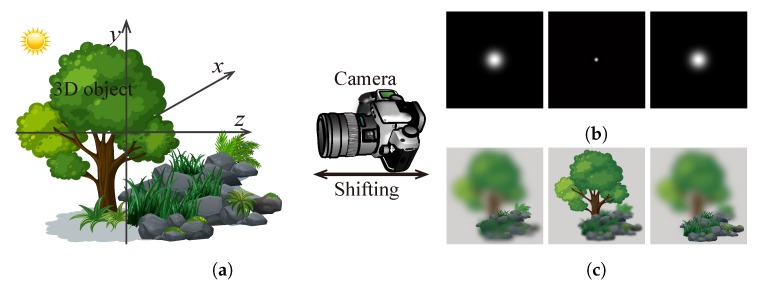
Scheme of focal sweeping capture (**a**); and a sample of the captured images of a point source (**b**) and a 3D scene (**c**).

In the focal plane sweeping capturing system, usually, only a camera with no other optical components is used, as Figure 7 shows. The captured image $I_{i}\left(r\right)$ is the convolution between the image of the object and the point spread function (PSF) of the system at the corresponding plane [52]. $I_{i}\left(r,z_{c}\right)$, a captured image at a plane of $z_{c}$, thus can be expressed as

(4) $$\begin{array}{c} I_{i}\left(r,z_{c}\right)=\int O(r,z)⊗h_{i}\left(r,z_{c}-z\right), \end{array}$$

where $h_{i}\left(r,z\right)$ is the PSF of an incoherent imaging system, and ⊗ is the 2D convolution operator. Since the aperture of a camera is usually circular, its PSF is approximately a 3D Gaussian distribution function which is symmetrical with respect to the focal plane along the optical axis [53,54]. Figure 7b shows a sample of several simulated 2D slices of the 3D PSF in a conventional camera imaging system, and Figure 7c shows several sample images captured at different planes.

Because of Equation (4), the early light ray field reconstruction algorithms from photographic images use image deconvolution, which is an inverse process of Equation (4) [55]. However, the blur kernel, which is related to the PSF, requires dense sampling to build, and thus could not give good results when the number of samples is limited. Subsequently, techniques involving the insertion of coded masks into a camera have been invented to obtain a higher resolution light ray field [17,56]. Although they achieve a better resolution than the lens array based techniques, they sacrifice light transmission because of the masks. In addition, they usually require solving a computationally intensive inverse problem, often with prior knowledge of the object scene. All of these problems limit their applications [36,57].

In recent years, focal stack imaging has attracted a lot of attention [58]. Focal stack is well known as a tool for extended depth of field photography [59] and fluorescence tomography [60]. Researchers have reported that it is also possible to reconstruct a light ray field from a multi-focus image stack [61,62,63]. The reason why a light ray field can be extracted from a focal stack is obvious, as the 3D information is stored in the stack. This can also be explained mathematically. It is due to the interchangeable property between light field and photographic images presented by Equations (1) and (4), which is fundamental to most light ray reconstruction techniques that are based on depth measurements. In such techniques, there is no need to mount or insert any additional optical elements to the camera, making it possible to capture a light ray field with commercial DSLR cameras.

To achieve focal plane shifting, we can shift both the camera and the object along the optical axis [62], rotate the focus ring of the camera [26], or modulate the PSF by optical components [64,65]. The camera shift can be controlled digitally [51,66] or mechanically [26,62]. The reconstructed light ray field has a high resolution comparable to the modern camera sensor because the images are not segmented by the sub-lens of the lens array. It can also be used for digital refocusing of the scene, synthesizing apertures of arbitrary shapes and sizes, estimating the structure of the scene, and reconstructing the 3D shapes [62,63].

In the following section, we give a review on these kinds of techniques, especially the light field reconstruction with back projection (LFBP) approach [62,63] and the light field moment imaging (LFMI) [26,67].

### 3.2. Light Ray Field Reconstruction by Back-Projection

Because of the interchangeable relationship between light ray field and 2D photographic images, the light field can be reconstructed from the depth measured photographic images directly by back-projection.

Suppose $I_{i}\left(r,z_{q}\right)$ is a photographic image taken at $z=z_{q}$, and *m* is the index number of one image in the photo stack. The total number of captured images is denoted as *Q*. With these captured images, the light field with the principal plane located at z=0 is calculated by using the back-projection algorithm [62]

(5) $$\begin{array}{c} L\left(r,\theta ,0\right)=\frac{1}{Q}\sum_{q=1}^{Q}I_{i}\left(r+z_{q}\theta ,z_{q}\right). \end{array}$$

**Figure 8:**
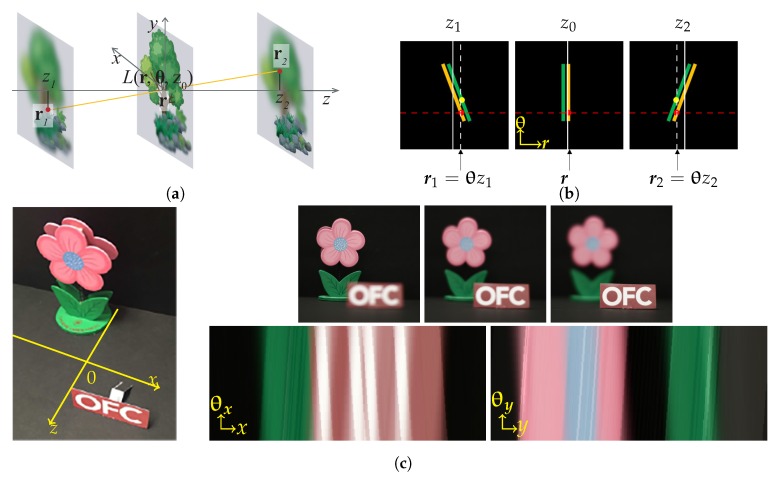
Principle of light field reconstruction with back projection (LFBP) represented in the spatial domain (**a**) and by Wigner distribution function (WDF) (**b**), and an example of the reconstructed epipolar plane images (EPIs) of a real 3D scene (**c**), adapted with permission from [66], Optical Society of America).

We name the light ray field reconstruction directly with this equation as LFBP I. Here, we neglect the magnification factor of the images. This is because the captured images can be aligned and resized easily with digital post-processing. Equation (5) can be explained by Figure 8 more intuitively. In Figure 8a, a light ray L(r,θ,0) with a propagation direction of θ contributes to two different positions in the defocus images, in front of and behind it, i.e., $r_{1}$ in the front image and $r_{2}$ in the rear image. The positions can be obtained by the projection angle and the depth position of these images by $r_{q}=r+z_{q}\theta$. Based on this fact, the radiance of the light ray can be obtained by the average value of the pixels on all of the depth images along the ray directions.

Figure 8b shows the corresponding epipolar plane image (EPI) explanation. Focal plane sweeping in spatial space corresponds to shearing of the EPI, with the degree of shearing reflecting the focal plane sweeping distance [62], in a way similar to the property of the Wigner distribution function (WDF) [68]. A ray with a fixed propagation angle corresponds to different shearing of the focused EPI; therefore, its radiance can be obtained by integrating over all the red points in the three EPI images, i.e., accumulation along the red dashed horizontal line. Figure 8c shows an example of the LFBP I technique, where the left image shows the 3D object scene, and the upper right images are the captured images, whereas the bottom right images are the EPIs of the reconstructed light field. Since the captured images are not segmented by lens arrays, the reconstructed light ray field shows a better angular and spatial resolution, as reflected by the EPIs in Figure 8c. The spatial image resolution is comparable to that of a conventional camera sensor. Note that the angular sampling of the light field calculated from the photographic images depends on the numerical aperture (NA) and the pixel pitch of the camera sensor, rather than the number of images captured along the optical axis.

As the EPIs shown in Figure 8c, the light field reconstructed with this approach has a severe noise problem [62,66,69]. The EPIs show overlap, and this phenomenon is very serious when the 3D scene is complicated. Chen et al. have analyzed this noise by giving the exact expression of the LFBP that relates to the depth measurements [51]:

(6) $$\begin{array}{c} L^{\prime }\left(r,\theta \right)=\sum_{q=1}^{Q}O(r+z_{q}\theta ,z_{q})+\sum_{q=1}^{Q}\int_{z\neq z_{q}}O(r+z_{q}\theta ,z)⊗h_{i}\left(r+z_{q}\theta ,z_{q}-z\right)dz. \end{array}$$

**Figure 9:**
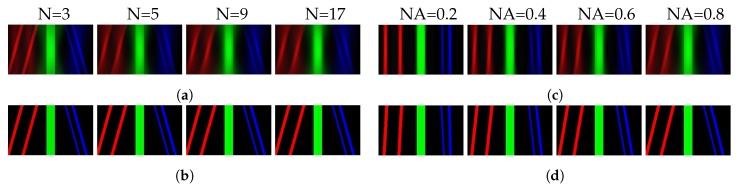
The reconstructed EPIs calculated from various number of depth images (**a**,**b**) and five depth images captured under various camera numerical apertures (NA) (**c**,**d**), by the conventional LFBP I (**a**,**c**) and denoised LFBP II (**b**,**d**), respectively, adapted with permission from [51], Optical Society of America.)

Since a 3D object O(r,z) can be discretized along the optical axis by $O\left(r,z\right)\approx \sum_{n=1}^{N}O\left(r,z_{n}\right)$, where *N* is the slice number, Equation (3) can be rewritten as $L\left(r,\theta \right)=\sum_{n=1}^{N}O(r+z_{n}\theta ,z_{n})$. Therefore, when *Q* in Equation (6) approaches *N*, the first term approaches Equation (3), which corresponds to the discrete approximation of the 3D objects’ light field. When *Q* is much smaller than *N*, it is equivalent to axially sampling the object insufficiently, which affects the depth resolution of the reconstructed light field. The second term of Equation (6) is noise. Obviously, it is the accumulation of the defocus noise induced by the images of the object slices which are out of focus. From this equation, we can see that there are two main parameters affecting the noise: the number of depth images and the PSF of the camera. The PSF is related to the f-number of the camera, i.e., the NA. As the second term of Equation (6) shows, for the LFBP technique, smaller NA and fewer images produce a higher quality reconstructed light ray field. However, to maintain the depth resolution of the reconstructed light field, the number of the captured images should be large enough. This mutual constraint property makes it difficult to get a high-quality light field with the conventional LFBP technique. This can be observed from the original paper [62] and the EPIs of Figure 9a,c. Figure 9a shows the EPIs of the reconstructed light field vs. the number of depth measurements, and Figure 9c shows the results vs. different camera NAs, respectively, when the number of measurements is 5. The noise of the EPIs gets worse with an increasing number of measurements and camera NAs. In Figure 9a, the useful signal is submerged when the number of measurements is large enough, which can be seen from the rightmost image of Figure 9a. Figure 9c shows that correct EPIs cannot be reconstructed with a very small camera NA, and a large camera also induces signals to be submerged. 

Based on the fact that (1) each point on the 3D object only focuses at one plane in the depth images, and (2) the largest amount of changes of the depth images indicates the clearest image location, the defocus noise can be eliminated by preprocessing the depth images before light field reconstruction [51]. The quality of the reconstructed light field is improved a lot, as the EPIs shown in Figure 9b,d. The number of the captured images and camera NAs does not affect the reconstruction after defocus noise elimination. Similarly, more sophisticated depth map calculation algorithm with sequential layered EPI calculation can be applied to achieve more realistic light field reconstruction [63,70]. One method is akin to restructuring the 3D model firstly. Then, represent it with Equation (3). Hence, it is very dependent on the depth map calculation algorithm. For comparison, name the above two improved LFBP techniques as LFBP II [51,63].

### 3.3. Iterative Light Ray Field Reconstruction Based on Back-Projection

In order to obtain a more realistic light ray field reconstruction with higher quality, iterative light ray field reconstruction based on back-projection (iLFBP) techniques has been reported [71,72]. These kinds of techniques are based on the fact of the interchangeable property between 2D camera images and the light ray field, which is indicated by Equations (1) and (5). Based on this imaging geometry, both Yin et al. and Liu et al. have reported an iterative light ray field reconstruction technique from focal stack measurements [71,72]. Their methods share the same concept.

**Figure 10:**
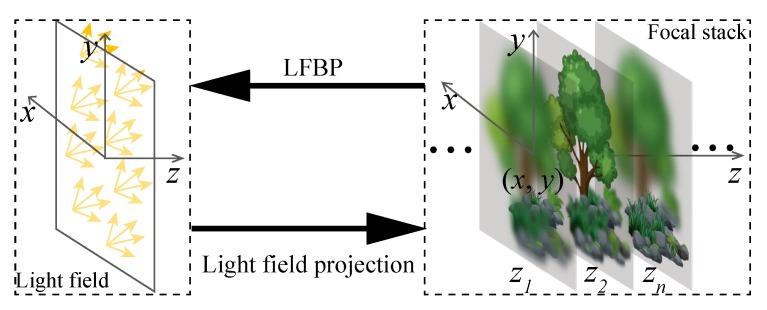
Scheme of iterative light ray field reconstruction based on back-projection (iLFBP).

In iLFBP, there is a projection bounce between the 2D focus stack images and the light ray field. For each bounce, a constraint related to the NA of the camera is applied. This is akin to the famous iterative phase retrieval algorithms that we will introduce in Section 4.1. Instead of using the LFBP I, a more sophisticated filtered back-projection algorithm [72] or a Landweber iterative scheme [71] is used to project measured focal stack images to a light ray field. This can make the best use of the structural information of the focal stack, as Figure 10 shows.

Since this kind of technique does not rely on a preprocessing or the complex nonlinear depth estimation process like in LFBP II, it is not affected by the accuracy of the depth estimation. The experiments show the advantages of these kinds of techniques in accuracy, reduced sampling, and occluded boundaries. However, as with all the other iterative based techniques, these kinds of techniques are heavily time-consuming.

### 3.4. Light Field Moment Imaging

As we illustrated in the previous context, 3D information is carried by the photographic stack images. Even though the light ray field can be reconstructed by LFBP I and LFBP II, the depth resolution is closely related to the axial sampling induced by the depth measurements. Although the iLFBP shows the possibility to use fewer images, it still requires quite a lot.

Motivated by the fact that light energy flow along the optical axis is reflected by the sweeping captured images, Orth and Crozier [26] have found that the angular moment of light rays satisfies a Poisson equation:

(7) $$\begin{array}{c} \frac{\partial I_{i}\left(r,z\right)}{\partial z}=-\nabla_{⊥}\;\cdot \;\nabla U\left(r,z\right), \end{array}$$

while ∇U is the product of the intensity with the light ray’s first angular moment M(r) at the position of (r,z), which is determined by intensity deviation of two [26] or more [67,73] images obtained at different focus distances. The angular moment is then used to reconstruct 3D perspective views of a scene by [26]

(8) $$\begin{array}{cc} L(r,\theta ,z) & =I_{i}\left(r,z\right)\exp\left\{-\frac{{\left[\theta -M\left(r\right)\right]}^{2}}{\sigma^{2}}\right\}\\ & =I_{i}\left(r,z\right)\delta \left[\theta -M\left(r\right)\right]⊗G\left(\theta ,\sigma \right), \end{array}$$

where G(θ,σ) is the Gaussian function with standard deviation σ, which equals the NA of the system. This is based on the fact that the angular moment of the light rays satisfy Gaussian function [26], and can be modified properly when the camera used in the capture has a different aperture shape.

**Figure 11:**
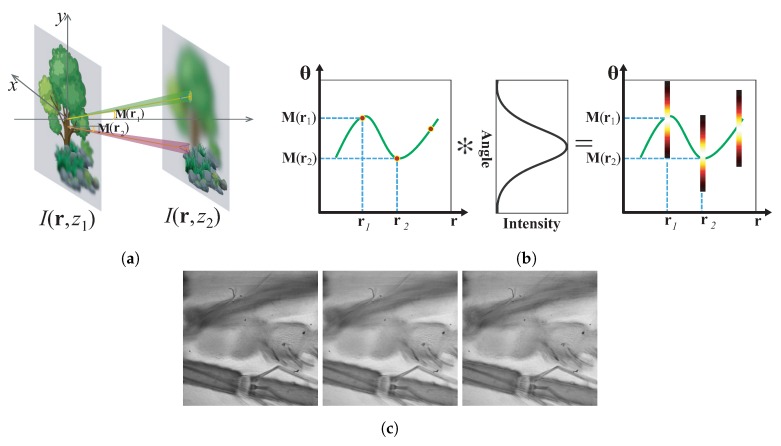
Principle of Light field moment imaging (LFMI) represented in the spatial domain (**a**) and WDF (**b**), and one example of different view images reconstructed by it (**c**), adapted with permission from [64], Optical Society of America).

The theory of LFMI can be explained more intuitively by Figure 11. Figure 11b represents the LFMI calculation process in one-dimensional (1D) EPI expression. The estimated angular moment represents the average light ray propagation direction at each spatial position, as shown in Figure 11a, thus can be represented as a curve in 1D EPI, as the left image in Figure 11b shows, which corresponds to $I_{i}\left(r_{n}\right)\delta \left[\theta -M\left(r_{n}\right)\right]$ in Equation (8). The final calculated EPI (Right image in Figure 11b) is the convolution between the angular moment and the Gaussian PSF (Center image in Figure 11b). It can be seen that the final EPI is mainly determined by the angular moment, whose accuracy affects the reconstructed light field the most.

Light field moment imaging has a counterpart in the wave-optics regime, the transport of intensity equation (TIE), which is a very effective tool for non-interferometric phase retrieval [74]. The key of LFMI is to obtain the first angular moment by solving a Poisson equation that is similar with TIE [26]. Thus, performance is very critical to the axial spacing between adjacent measurement planes [75,76]. Therefore, this axial spacing should be chosen carefully according to the object’s characteristics in order to obtain a good estimation of intensity derivative [26]. The estimation accuracy can be further improved with multiple images [67,73]. This issue is precisely identical to the noise-resolution tradeoff in TIE, which will be discussed in detail in Section 4.2.3.

It should also be mentioned that, although LFMI provides a new concept for light ray field reconstruction from depth measurements, it cannot reconstruct a complete light ray field, but some perspective view like images. However, it provides a new view on depth measurement based light ray field reconstruction, and is still useful for viewing a 3D shape of small objects to some extent. More detailed discussions about the relationship between FLMI and TIE can be found in Section 5.2.

### 3.5. Issues

In this section, we have introduced techniques of light ray field calculation from a series of depth images. Not being segmented by an array device, the spatial resolution is only limited by the camera NA as in conventional photography. As these methods do not require any special equipment like lens array or code masks, they are easy to implement. However, they have their issues. Table 1 shows the comparison of these techniques. LFBP based methods can reconstruct the light ray field with defocus noise [62], which can be reduced by preprocessing [51,63] or iterative approaches [71,72]. However, iteration makes the digital reconstruction time-consuming. LFMI [26] reconstructs images by estimating the first angular moment of the light rays instead of an exact light ray field with at least two depth measurements. More exact reconstruction may need more depth measurements [67].

**Figure 12:**
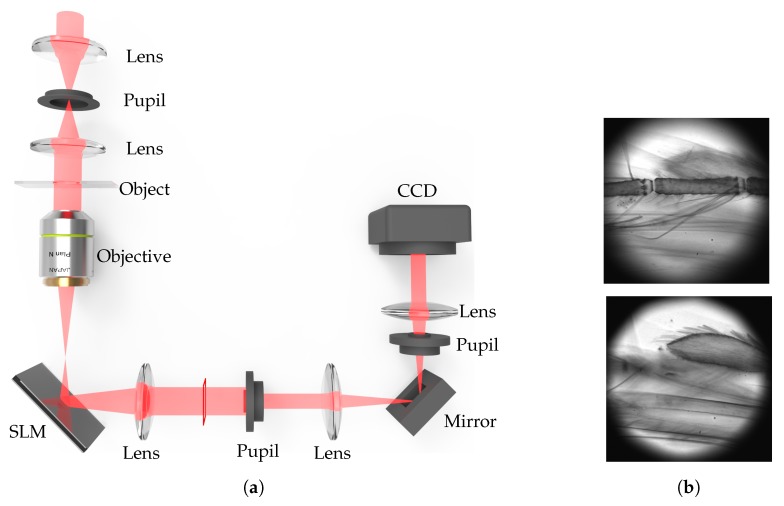
System setup (**a**) and two of the captured out of focus images (**b**) of the fast light ray field acquisition with point spread function (PSF) modulation (Adapted with permission from [64], Optical Society of America). SLM: spatial light modulator; CCD; Charge-coupled device.

The most common problem of the LFBP techniques is the depth resolution that is inherited from the depth measurement approach. This requires a large amount of measurements when the object has a large depth. Consequently, this is very time-consuming and requires fine and stable mechanical alignment in the capture process [51,62,63,72]. Therefore, Wang et al. has reported an efficient light-field acquisition technique by using a spatial light modulator (SLM) to obtain defocussing instead of mechanical translation [64]. This technique can achieve fast data acquisition and is free from mechanical instability. The modulation is implemented in a microscopy imaging system, as Figure 12 shows. With this system, the time cost for capturing a large amount of focal plane sweeping images is efficiently reduced. In addition, the accuracy of the captured images is increased because there is no mechanical movement during the capture process. This technique may also be used to achieve other types of PSF distribution functions rather than Gaussian. In this case, the Gaussian distribution function in the LFMI equation should be modified to the corresponding PSF function. The microscopic imaging system can also be extended to conventional digital imaging system by using an electrically tunable lens [78] for colorful imaging.

## 4. Wavefront-Based Light Field Imaging from Depth Measurements

Phase retrieval based wavefront reconstruction techniques do not require any reference beam. Generally, one or several diffracted intensity images and some post digital image processing are needed. These kinds of techniques are usually implemented by either iterative or deterministic approaches. Iterative phase retrieval techniques are based on the Gerchberg–Saxton (GS) method [79], and the deterministic approach usually means the TIE [74,80,81]. In the following, we review these two types of phase retrieval sequentially.

### 4.1. Iterative Phase Retrieval

**Figure 13:**
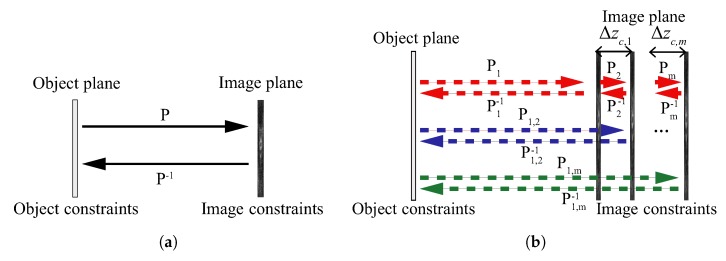
Iterative phase retrieval with (**a**) one single and (**b**) multiple depth measurements.

Almost all of the iterative phase retrieval techniques are based on the GS [79] and Yang–Gu (YG) [82] algorithms, which use prior knowledge of the object as constraints [18]. In the GS method, bounces between in-focus and Fourier domain images are performed, as shown in Figure 13a, *P* and $P^{-1}$ are a pair of wave propagation operators. At each step, an estimation of the complex-field is updated with measured or a priori information of the object [18,83,84]. In this case, the accuracy of the phase retrieval is an issue. Phase solutions with these techniques are not unique, but they are likely to be correct [85]. In addition, the stagnation of the iteration and the local minimal problem have limited its application. Many techniques have been proposed for lessening the solution error [86,87] since this technique was invented.

Fresnel transformed images can also be used instead of Fourier transformed images [88], i.e., *P* and $P^{-1}$ can be either Fourier transform or Fresnel transform. The Fresnel transformation between the object and measurement planes instead of Fourier transform was a milestone. This makes the iterations not only be confined between object and image plane, but among multiple diffracted intensity images. The iteration among the object and the measurements can have many combinations, as Figure 13b shows. Bounces can be performed for all of the planes in one loop through the path of $P_{1}\to P_{2}\cdots P_{m}\to P_{m}^{-1}\cdots \to P_{2}^{-1}\to P_{1}^{-1}$, or only be performed between object plane and each image plane sequentially through the path of $P_{1}\to P_{1}^{-1}\to P_{2}\to P_{2}^{-1}\cdots P_{m}\to P_{m}^{-1}$. It can even be performed only among the measurements. As a result, the prior knowledge about the object became unnecessary. Multiple measurements have improved the accuracy of the phase retrieval and the unnecessary object prior requirement, and result in a broader application of iterative phase retrieval [89,90,91].

In the past decades, research about the iterative algorithm, defocus of the measurements, light modulation and multiple measurements have been fully studied. Fienup has reported on the gradient decent search (GDS) algorithm and hybrid input-output (HIO) algorithm successively [18], where the HIO has proven to be very effective and has been widely used up to now [92,93].

Besides the improvement on the algorithm, the required properties of the measurements, which represent the image constraints, have also been studied. It has been demonstrated that the accuracy of the phase reconstruction is affected by the defocus amount of the measured images [88,94], which is object-dependent. Large propagation distance usually produces better diffraction contrast and thus makes these techniques work better for X-ray imaging [95,96]. A known illumination pattern used as a constraint can eliminate the prior knowledge requirement [90]. A random amplitude mask [97] or random phase plate [98,99] used to modulate the illumination requires less iterations for reconstruction because the low frequencies of the object being transformed to fast varying high frequencies. However, an amplitude mask results in the diminution of light energy, and a phase plate requires difficult fabrication.

Multiple measurements can also be regarded as improving the object constraint to some extent. Because multiple measurements with variations in depth detect spatial frequencies at different sensitivities [89,100,101,102,103], they can also improve the resolution of the reconstruction. Multiple depth measurements can be produced by capturing intensity images under illumination with different wavelengths at a single position [104], or under a single beam illumination with different foci [89], translating an aperture transversely [100]. If image quality is a concern, other types of multiple measurements, such as off-axis multiple measurements of synthetic aperture (SA) [105,106], ptychographic technique [105,106,107,108], and structured illumination [109,110] have been proven to be more efficient in image quality.

The significant amount of the data carried by multiple measurements makes the phase retrieval very robust and rather stable to noise [50]. It is obvious that more measurements result in higher quality reconstructions [111]. However, capturing more intensity images requires more movement steps of the camera or the object, which makes the captured images sensitive to small misalignments in the experimental setup, thus the noise induced to the captured intensity images becomes more serious. In addition, the time-consuming nature of the capture makes it not capable for use in dynamic object or real-time applications. To improve the multiple measurement capture [102], the devices such as beam splitters [50], SLM [112,113], and deformable mirror (DM) [114] were used to achieve single-shot/single-plane measurements. However, the beam splitter causes light attenuation, and the use of SLM or DM sacrifices the simplicity of the experimental setup. All of these approaches require additional optical components and involve complicated post digital processes. Therefore, algorithms based on multiple measurements have also been developed on the other hand [103].

The typical iterative phase retrieval techniques are summarized in Table 2. Despite the limitations of each technique, the iterative phase retrieval remains a popular technique for wavefront reconstructions due to the fact that the optimal transfer function is object-dependent and due to the simplicity of its implementation. However, iterative phase retrieval based on scalar diffraction theory [52] works under coherent illumination, which limits its application. TIE, which is presented in the next section of 4.2, has been proved to be a compensation to an iterative phase retrieval technique.

### 4.2. Transport of Intensity Equation

Another important non-interferometric single-beam phase retrieval approach is called TIE. It was originally derived by Teague [20] from the Helmholtz equation under paraxial approximation more than 30 years ago, when its main application fields were adaptive optics [115], transmission electron microscopy (TEM) [116], X-ray imaging [117] and neutron radiography [118]. Recently, technological advancements in optical microscopy and digital signal processing have brought TIE back to the forefront of quantitative phase microscopy [119,120,121,122] and 3D depth imaging [26,64,123]. The TIE specifies the relationship between object-plane phase and the first derivative of intensity with respect to the optical axis in the near Fresnel region, yielding a compact equation which allows direct recovery of phase information [20]

(9) $$\begin{array}{c} -k\frac{\partial I(r)}{\partial z}=\nabla \;\cdot \;\left[I(r)\nabla \phi (r)\right], \end{array}$$

where *k* is the wave number 2π2πλλ, and r is the position vector representing the transverse spatial coordinates (x,y). ∇ is the gradient operator over r, which is normal to the beam propagation direction *z*. I(r) is the intensity, located without loss of generality at the plane z=0, and ϕ(r) is the phase to be retrieved. Expanding the right-hand side (RHS) of Equation (9), one obtains

(10) $$\begin{array}{c} -k\frac{\partial I(r)}{\partial z}=\nabla I\left(r\right)\;\cdot \;\nabla \phi \left(r\right)+I\left(r\right)\nabla^{2}\phi \left(r\right). \end{array}$$

In the above expression, the first term on RHS is called prism term, which stands for the longitude intensity variation due to the local wavefront slope. The second term on RHS is called lens term representing the intensity variation caused by the local wavefront curvature. It can be seen that TIE links the longitudinal intensity derivative with the slope and curvature of the wavefront which produces the change in intensity as the wavefront propagates.

#### 4.2.1. Solutions to Transport of Intensity Equation

Transport of intensity equation is a second order elliptic partial differential equation for the phase function, and solving this equation does not appear to be difficult. Supposing I(r)>0 within enclosure $\bar{\Omega }$ and with appropriate boundary conditions (defined on the region boundary ∂Ω), the solution to the TIE is known to exist and be unique (or unique apart from an arbitrary additive constant) [80], i.e., the phase ϕ(r) can be uniquely determined by solving the TIE with the measured intensity I and the axial intensity derivative ∂I/∂z. The TIE is conventionally solved under the so-called “Teague’s assumption” so that the transverse flux I∇ϕ is conservative and can be fully characterized by a scalar potential ψ (an auxiliary function):

(11) $$\begin{array}{c} \nabla \psi =I\nabla \phi . \end{array}$$

Then, the TIE can be converted into the following two Poisson’s equations:

(12) $$\begin{array}{c} -k\frac{\partial I}{\partial z}=\nabla^{2}\psi , \end{array}$$

and

(13) $$\begin{array}{c} \nabla \;\cdot \;\left(I^{-1}\nabla \psi \right)=\nabla^{2}\phi . \end{array}$$

Solving these two Poisson’s equations is straightforward mathematically, and several numerical solvers have been proposed, such as the Green’s function method [20,124], the multi-grid method [125,126], the Zernike polynomial expansion method [127,128], the fast Fourier transform (FFT) method [81,125,128,129], the discrete cosine transform (DCT) method [130,131], and the iterative DCT method [132].

Techniques about TIE solution, boundary conditions, phase discrepancy, axial derivative are compared in Table 3.

**Figure 14:**
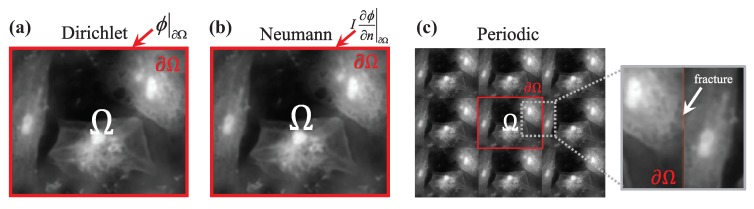
Three typical boundary conditions used in TIE solvers: (**a**) Dirichlet boundary conditions (need to know the phase value at the boundary); (**b**) Neumann boundary conditions (need to know the phase normal derivative at the boundary); and (**c**) periodic boundary conditions (assume the object is periodically extended at the boundary).

Despite its mathematical well-possessedness, the rigorous implementation of the TIE phase retrieval tends to be difficult because the associated boundary conditions are difficult to measure or to know as a priori. Figure 14 shows three typical boundary conditions used in TIE solvers: Dirichlet boundary conditions, Neumann boundary conditions, and periodic boundary conditions. Since the phase function is exactly the quantity to be recovered, its value ${\phi |}_{\partial \Omega }$ (for Dirichlet boundary condition) or normal derivative ${I\partial \phi /\partial n|}_{\partial \Omega }$ (for Neumann boundary condition) at the boundary cannot be known in advance before taking any measurements. To bypass the difficulty, many researchers have tried to solve TIE directly without explicitly imposing the boundary conditions [128,133,134,147]. Coincidentally, all the efforts aim to find some ways to nullify the overall energy transfer across the region boundary, making boundary conditions unnecessary

(14) $$\begin{array}{c} {\;\int \int \;}_{\Omega }\frac{\partial I(r)}{\partial n}dr=0. \end{array}$$

**Figure 15:**
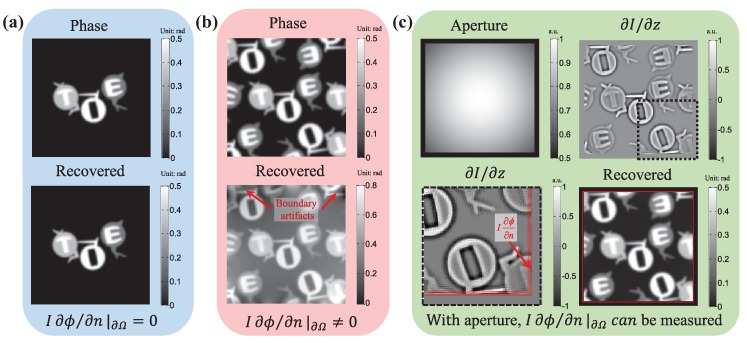
Phase retrieval simulations for different types of objects: (**a**) an isolated object located in the central field of view (FOV) (FFT-based solver gives accurate reconstruction); (**b**) a complex object extending outside the image boundary (FFT-based solver produces large boundary artifacts); and (**c**) DCT solver with a hard aperture (the inhomogeneous boundary conditions can be measured at the boundary, which produces accurate phase reconstruction even if the object is located at the aperture boundary).

One simple and common way to satisfy this condition is to let the measured sample be isolatedly placed in the center of the camera field of view (FOV), surrounded by an unperturbed plane wave (the phase is “flat” at the boundary of the FOV), in which case the energy (intensity) conservation is fulfilled inside the FOV at different image recording locations, as shown in Figure 15a. Then, one can safely define some simplified boundary conditions, e.g., the homogeneous Dirichlet conditions (zero phase changes at the boundary ${\phi |}_{\partial \Omega }=C$, where *C* is a constant), the homogeneous Neumann boundary conditions (constant phase at the boundary ${I\partial \phi /\partial n|}_{\partial \Omega }=0$), or the periodic boundary conditions (the phase at the boundary repeats cyclically). In this case, the most popular FFT based TIE solver [81,125,128,129,147] works well because it implies periodic boundary conditions due to the cyclic nature of the discrete Fourier transform. Nevertheless, this configuration is rather restrictive and does not reflect general experimental conditions. When the actual experimental condition violates those imposed assumptions, e.g., in wavefront sensing (non-fact phase at the boundary) or objects extending outside the image boundary, as shown in Figure 15b, severe boundary artifacts will appear, and they seriously affect the accuracy of the phase reconstruction [133,134,147].

To bypass the difficulty in obtaining real boundary conditions, Gureyev and Nugent [128] suggested another way to eliminate the need of boundary conditions by considering the special case that the intensity is positive inside the domain Ω but strictly vanishes at the boundary (so that ${I\partial \phi /\partial n|}_{\partial \Omega }=0$). Alternatively, without any additional requirement about the test object and experimental conditions, Volkov et al. [133] proposed a pure mathematical trick to nullify the energy flow across the boundary through appropriate symmetrization of input images. However, it assumes that there is no energy dissipation through the image boundary for any objects, which is generally not physically grounded. To summarize, *‘without boundary value measurements’ does not mean that the TIE can be solved without imposing any boundary conditions, or more exactly, we have to confine our measured object or experimental configuration to certain implicit boundary conditions.*

For more general cases, as shown in Figure 15b, the energy inside the FOV is not conserved, as energy “leak” occurs at the FOV boundary while the recording distance is being changed. In this case, inhomogeneous boundary conditions are thus necessary for the correct phase reconstruction based on TIE. Zuo et al. [130] addressed the solution of the TIE in the case of *inhomogeneous Neumann boundary conditions* under nonuniform illuminations. By introducing a hard aperture to limit the wavefield under test, as shown in Figure 15c, the energy conservation can be satisfied, and the inhomogeneous Neumann boundary values ${I\partial \phi /\partial n|}_{\partial \Omega }$ are directly accessible around the aperture edge. In the case of *rectangular aperture*, the DCT can be used to solve the TIE effectively and efficiently, which has been well demonstrated in application of microlens characterization [131]. Huang et al. [132] further extended the DCT solver to an *arbitrarily shaped aperture* by an iterative compensation mechanism. Recently, Ishizuka et al. [148,149] successfully applied the iterative DCT solver to recover the additional phase term corresponding to the curvature of field on the image plane in TEM.

#### 4.2.2. Phase Discrepancy and Compensation

Another notable issue regarding the solution of the TIE is “phase discrepancy” resulting from the introduction of the Teague’s auxiliary function [20], which suggests that the transverse flux is conservative so that a scalar potential ψ exists that satisfies Equation (11). However, it is important to note that the Teague’s auxiliary function does not always exist in practical situations since the transverse energy flux may not be conservative, and consequently it would produce results that would not adequately match the exact solution [150]. This problem was first pointed out by Allen et al. [125] in 2001. Ten years later, Schmalz et al. [150] made a detailed theoretical analysis on this problem based on the Helmholtz decomposition theorem and decomposed the transverse flux in terms of the gradient of a scalar potential ψ and the curl of a vector potential η:

(15) $$\begin{array}{c} I\nabla \phi =\nabla \psi +\nabla \times \eta . \end{array}$$

**Figure 16:**
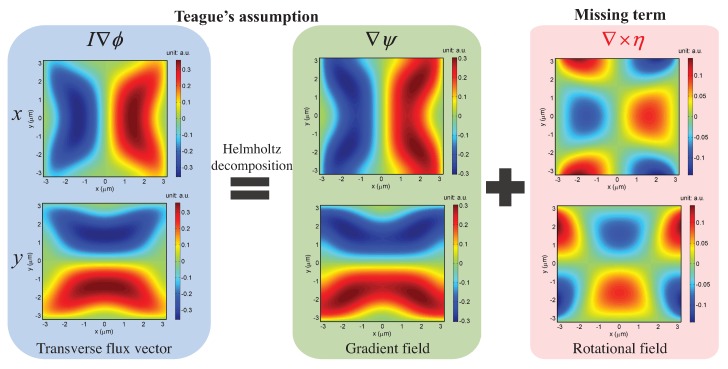
Helmholtz decomposition of the transverse flux field. The *x* and *y* components of the vector fields are shown in the first row and the second row, respectively. The term ∇×η is missing in Teague’s assumption.

Compared with Equation (12), it is plain to see that the term ∇×η is ignored in Teague’s assumption, making a silent hypothesis that the transverse flux is irrotational (see Figure 16). In 2014, Zuo et al. [135] examined the effect of the missing rotational term on phase reconstruction, and derived the necessary and sufficient condition for the validity of Teague’s assumption:

(16) $$\begin{array}{c} \nabla I^{-1}\times \nabla^{-2}\left[\nabla \;\cdot \;(\nabla I\times \nabla \phi )\right]=0. \end{array}$$

Equation (16) shows that, if the in-focus intensity distribution is nearly uniform, the phase discrepancy resulting from the Teague’s auxiliary function is quite small $\left(\nabla I^{-1}\times \nabla^{-2}\left[\nabla \;\cdot \;\left(\nabla I\times \nabla \phi \right)\right]\approx 0\right)$. However, when the measured sample exhibits strong absorption, the phase discrepancy may be relatively large and cannot be neglected [150,151]. To compensate the phase discrepancy owing to Teague’s assumption, Zuo et al. [135] further developed a simple Picard-type iterative algorithm [135], in which the phase is gradually accumulated until a self-consistent solution is obtained. Within two to four iterations, the phase discrepancy can be reduced to a negligible level, and the exact solution to the TIE can be thus obtained.

#### 4.2.3. Axial Intensity Derivative Estimation

**Figure 17:**
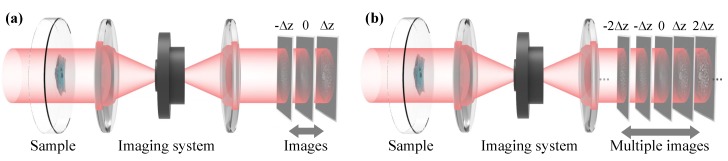
Typical experimential setup for TIE phase retrieval. (**a**) conventional three-plane TIE; (**b**) multi-plane TIE. The through-focus intensity stack can be acquired by moving either the object or the image sensor.

From the previous section, we know that, in order to solve the TIE, one needs to know the intensity I and axial intensity derivative ∂I/∂z. Experimentally, the in-focus intensity I is easy to obtain. However, the intensity derivative along the optical axis cannot be directly measured. Conventionally, it is estimated by a finite difference between two out-of-focus images, recorded symmetrically about the in-focus plane with ±Δz defocus distances [20], as illustrated in Figure 17a:

(17) $$\begin{array}{c} \frac{\partial I(r)}{\partial z}\approx \frac{I_{\Delta z}\left(r\right)-I_{-\Delta z}\left(r\right)}{2\Delta z}. \end{array}$$

Mathematically, this approximation is valid in the limit of small defocus distances, where the error is the second order of the focus distance if the data are noise-free. However, experimentally the derivative estimate will become quite unstable when the distance Δz is too small because of the noise and quantization error [141]. On the other hand, increasing the two-plane separation Δz provides better signal-to-noise ratio (SNR) in the derivative estimate, but the breakdown of the linear approximation induces nonlinearity errors, which results in loss of high frequency details [139]. Thus, a compromise has to be made where Δz is chosen to balance the nonlinearity error and the noise effect [152]. Specifically, the optimal Δz is dependent on both the maximum physically significant frequency of the object and the noise level [152,153]. However, a priori knowledge about these two aspects is difficult to be known in advance.

To overcome this trade-off, there has been an increased effort to improve the intensity derivative estimate by utilizing information recorded in multiple defocused planes [136,137,138,139,140,141,142,143,144,145,146]. As illustrated in Figure 17b, with more intensity measurements $I_{j\Delta z}\left(r\right),j=-n,\ldots ,-1,0,1,\ldots ,n$, the longitudinal intensity derivative can be represented by their linear combination:

(18) $$\begin{array}{c} \frac{\partial I(r)}{\partial z}\approx \sum_{j=-n}^{n}\frac{a_{j}I_{j\Delta z}\left(r\right)}{\Delta z}. \end{array}$$

Thus, it offers more flexibility for improving the accuracy and noise resistance in derivative estimation. Numerous finite difference methods have been proposed such as high-order finite difference method [136,138,139,154], noise-reduction finite difference [137,155], higher order finite difference with noise-reduction method [140], and least-squares fitting method [139]. The only difference in these multiple planes derivative estimation methods lies in the coefficients $a_{j}$ in Equation (18), and it has been found that all these methods can be unified into the Savitzky–Golay differentiation filter (SGDF) [156,157,158] with different degrees if the finite difference (Equation (18)) is viewed from the viewpoint of digital filter [141]. Different from these finite difference methods with a fixed degree, methods that decompose the phase in the spatial frequency domain and estimate each Fourier component of the *z* derivative with an appropriately chosen finite difference approximation are particularly effective because they can balance the effects of noise and diffraction induced nonlinearity over a wide range of spatial frequencies [141,142,143,144]. For example, the optimal frequency selection (OFS) scheme proposed by Zuo et al. [141] uses a complementary filter bank in spatial frequency domain to select the optimal frequency components of the reconstructed phases based on SGDF with different degrees to produce a composite phase image. Martinez-Carranza et al. [143] extended the idea of multi-filter and frequency selection to accommodate the conventional three-plane TIE solver. Jenkins et al. [145] extended the basic principles of the multi-filter phase imaging to the important practical case of partially spatially coherent illumination from an extended incoherent source. Falaggis et al. [75] found that the optimum measurement distances obtained by multi-plane phase retrieval form a geometric series that maximizes the range of spatial frequencies to be recovered using a minimum number of planes. This strategy has been successfully implemented in the Gaussian Process regression TIE (GP-TIE) [146] and optimum frequency combination TIE (OFC-TIE) approaches [144], providing high accuracy of phase reconstruction with a significantly reduced number of intensity measurements.

**Figure 18:**
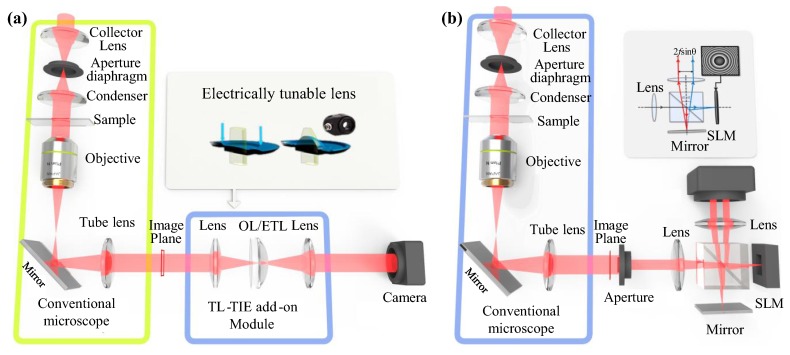
Advanced experimential setups for dynamic TIE phase imaging, which can be implemented as a simple add-on module to a conventional microscope. (**a**) electrically tunable lens based TIE microscopy system (TL-TIE) (adapted with permission from [78], Optical Society of America); (**b**) single-shot TIE system based on a SLM (SQPM); adapted with permission from [122], Optical Society of America).

Though multi-plane TIE approaches can solve the trade-off between noise and spatial resolution in conventional three-plane TIE, they require much more intensity measurements at different defocus distances. Manual adjustment or mechanical translation inevitably slows down the data acquisition speed, precluding real-time phase imaging of the dynamic process. This issue is basically the same as the one encountered in multi-plane iterative phase retrieval algorithms. Techniques based on multi-wavelength illumination [159,160], electrically tunable lens [78,161], SLM [122,162], and tilted flow cytometry [163] have been reported to yield a fast and tunable focusing scan of the sample, enabling dynamic TIE phase imaging by eliminating the need for any manual or mechanical operations and synchronization of multiple cameras. Furthermore, as shown in Figure 18, some techniques [78,122,161] can be implemented as an add-on module to a commercial microscope, enabling new features to quantitative phase imaging: diminished speckle effects due to partially coherent illumination, and multimodal investigation potential due to overlaying with other modalities of the microscope (e.g., fluorescence, differential interference contrast (DIC), phase contrast). More recently, it has been found that the shape of the illumination aperture has a significant impact on the lateral resolution and noise sensitivity of TIE reconstruction [164,165,166,167], and by simply replacing the conventional circular illumination aperture with an annular one, high-resolution low-noise phase imaging can be achieved by using only three-plane intensity measurements [164,165].

### 4.3. Discussion

Iterative phase retrieval and TIE are both important propagation-based phase imaging techniques, in which the phase contrast is formed by letting the wavefield propagate in free space after interaction with the object. TIE is valid under paraxial approximation and in the limit of small propagation distances (near-Fresnel region). The iterative phase retrieval is less restrictive, which does not rely on paraxial approximation, and is valid for both small and large propagation distances. However, it can sometimes exhibit slow convergence and stagnation problems described in Section 4.1. TIE is based on solving the intensity transport along the optical wave propagation, which does not explicitly resort to the scalar diffraction theory and does not need iterative reconstruction (deterministic). In addition, it generally requires less intensity measurements than iterative phase retrieval, and can directly recover the absolute phase without requiring phase unwrapping [120,168,169]. Thus, the complexity associated with the 2D phase unwrapping can be bypassed. More importantly, as will be introduced in Section 5.2, TIE is still valid for partially coherent illumination [81,170,171,172,173], making it suitable for use in a bright-field microscope [119,120,121,122]. However, TIE has its inherent limitations as a tool for quantitative phase retrieval, such as the requirement of small defocus, paraxial approximation, and the inaccurate solutions induced by Teague’s assumption and inappropriate boundary conditions.

Combination of the TIE and iterative process has been proved to be particularly effective and able to achieve complementary advantages [159,174,175,176,177,178]. For example, deterministic phase retrieval can be used to provide a “coarse” initial phase, which is then refined by iterative methods, e.g., GS method to recover continuous and discontinuous phase in both near- and far-Fresnel region [159,175,177,178]. Since the TIE reconstructed phase provides a very good initial approximation of the true phase, the convergence of iterative methods can be significantly improved and the stagnation problems associated with the GS-type iterative algorithm can be effectively avoided.

## 5. Connection between Ray-Based and Wave-Based Light Field Imaging

Due to the close connection of the ray and wave optics [7], 3D imaging techniques based on these two theories have a lot in common. Yamaguchi regards the phase of a wavefront as a contribution to the resolution of light ray field [3]. This can be comprehended intuitively from Figure 2 to some extent. Direction of light rays are along the normal of the wavefront, which is kind of sparse sampling of the continuous wavefront in the physical world.

The close connection between wavefront and light rays is reflected by many techniques such as Shack–Hartmann wavefront sensor, light ray field imaging with lens array, the TIE, and LFMI. There are also many techniques that take advantage of this fact such as synthesized hologram from multiple view images [179], hybrid approaches like holographic stereogram [180,181], and 3D Fourier ptychographic microscopy [182]. In the following sections, we show the close connection between wavefront-based and ray-based light field imaging in detail.

### 5.1. Shack–Hartmann Wavefront Sensor and Light Ray Field Camera

**Figure 19:**
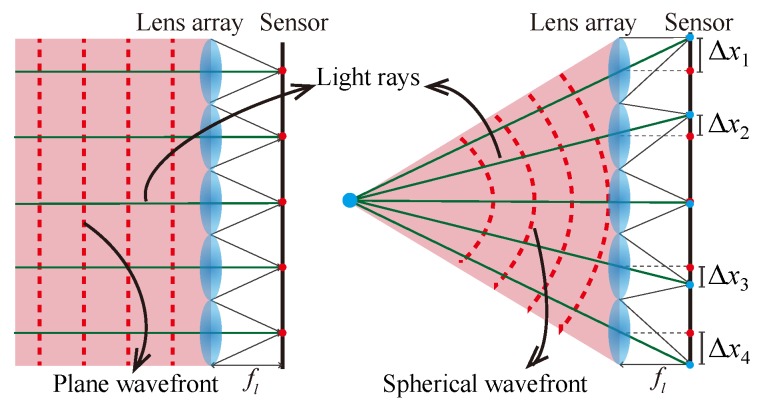
Shack–Hartmann wavefront sensor.

The Shack–Hartmann sensor is a well known wavefront sensing technique. In a Shack–Hartmann sensor, a lens array is placed in front of the camera sensor and the intensity images formed in the camera sensor are used to retrieve the income wavefront. Figure 19 shows the structure of the Shack–Hartmann sensor. When a wavefront enters it, there is a focal spot formed on the image sensor behind each individual lenslet. The location of each spot, noted as $\Delta x_{n}$, represents the phase gradient ($\Delta x_{n}/f_{l}$) of the wavefront at the corresponding sub-area. The overall wavefront can then be determined by the phase gradient across the entire wavefront.

People who are familiar with light ray field imaging with micro lens array [13] can easily find that they share a similar sensing setup: both insert a lens array in front of the camera sensor. This similarity of hardware design comes from the truth that light ray and wavefront are essentially equal to each other. For example, in Figure 19, the plane wavefront forms the same local point positions in the sensor, like the orthographic projection image of the light ray field [183]. The spherical wavefront, which introduces different local positions of the point image, is similar to the perspective view images of a point object. The position displacements correspond to view the parallax in light field imaging. Ko et al. have analyzed the relationship between the Shack–Hartmann sensor and lenslet array based light ray field imaging in detail [184]. A comparison under the scenario of wave-optics will be given in the following section.

### 5.2. Transport of Intensity Equation and Light Field Moment Imaging

As we mentioned in Section 4.2, the original TIE was derived from Helmholtz equation by Teague [20]. However, Teague’s TIE is under paraxial approximation and assumes a monochromatic, completely coherent beam. This assumption is not applicable to partial coherent wavefields. Optical coherence theory [7,52] is the formalism used to describe the field in this case, and it is formulated via a description that uses the 2nd order correlation functions of the field, such as the 4D mutual coherence function and cross-spectral density. However, the mathematics behind these quantities are quite complicated, making the results difficult to interpret. The alternate school of thought emerges from computer scientists who primarily deal with geometric optics. Generally, in an optical imaging system, all rays coming from a point of the object are focused into an image point at the conjugate location of the object point. Information of ray direction is lost, hence no perspective and depth information can be imaged. In geometric optics, the phase concept does not exist. Instead, the quantity interested is called the light field, as we introduced in Section 3 [30,185], which has information of both radiance and direction of light rays and could be related to the phase in wave optics. This is exactly the central issue of this section—what is the meaning of the term “phase" and how is it related to the light field?

**Figure 20:**
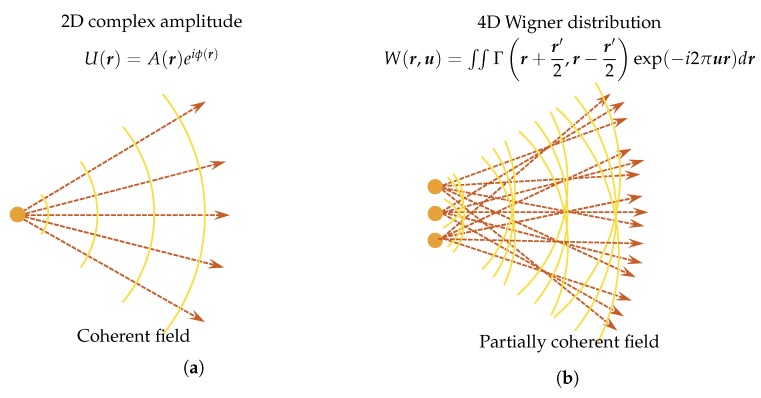
Schematic of a simplistic view of coherent field and partially (spatially) coherent field. (**a**) for coherent field, the surface of the constant phase is interpreted as wavefronts with geometric light rays traveling normal to them. It is fully described by 2D complex amplitude; (**b**) partially coherent field needs 4D coherence function, like the Wigner distribution, to accurately characterize its properties, like its propagation and diffraction. In addition, a partially coherent field does not have a well-defined phase, but rather a statistical ensemble of phases (spatial frequencies, propagation directions) at every position in space.

The definition of “phase” is clear for a coherent complex scalar wave, and it equals the argument of the field’s complex function. Such a perfect spatial and temporal coherent field has a wavefront corresponding to surfaces of the constant phase. In the geometric optics picture, the surface of the constant phase is the normal of the geometric light rays, as illustrated in Figure 20a. There is a direction coincidence between the phase surface and the time-averaged Poynting vector [81,170]. However, the light field representation in ray optics is inadequate to describe wave optical phenomena, such as interference, diffraction, and coherence effects. Moreover, for a non-coherent field, the coherent “phase” definition breaks down and we need a more general description of it.

As a quasi-probability distribution to bridge wave optics to ray optics, WDF, which is a phase-space distribution, has been introduced to study partially coherent fields [186]. The WDF is a spatial frequency-space description of a signal. This idea is akin to the concepts of rays’ angle and position description, as illustrated in Figure 20b. The WDF constitutes a rigorous wave-optical foundation for the radiometry theory with the possible negativity [187], and thus can visualize wave optical information like interference and diffraction effects. Compared with mutual coherence and cross-spectral density function, the WDF is able to represent a partially coherent beam in a more straightforward way. It is therefore desirable to have a simple mathematical phase-space TIE model, providing insights into the meaning of the term “phase” for a partially coherent field, and facilitate productive exchangeable ideas between the TIE and light field.

#### 5.2.1. Generalized Transport of Intensity Equation for Partially Coherent Field

Taking the phase-space theory as the starting point and based on the Liouville transport equation [186], Zuo et al. [173] derived the generalized TIE (GTIE) for the partially coherent field

(19) $$\begin{array}{c} \frac{\partial I(r)}{\partial z}=-\nabla \;\cdot \;\;\int \int \;\lambda uW_{\omega }\left(r,u\right)dud\omega , \end{array}$$

where u is the spatial frequency coordinate corresponding to r. $W_{\omega }\left(r,u\right)$ is the WDF of a given monochromatic component of the whole field with optical frequency of ω=c/λ, where *c* is the speed of light and λ is the wavelength. When the field is quasi-monochromatic, it can be regarded approximately as completely temporally coherent. Then, Equation (19) is simplified to the GTIE for partially spatially coherent fields [173],

(20) $$\begin{array}{c} \frac{\partial I(r)}{\partial z}=-\lambda \nabla \;\cdot \;\int uW(r,u)du. \end{array}$$

If we further limit the field to be completely spatially coherent, it can be fully represented by the 2D complex amplitude function $U\left(r\right)=\sqrt{I(r)}\exp\left[i\phi \left(r\right)\right]$, where ϕ(r) is the phase of coherent field, as shown in Figure 20a. From the perspective of time (space)-frequency analysis, we can consider the completely coherent field as a mono-component signal. The first order frequency moment of WDF is thus related to the phase gradient of the coherent field [188,189]:

(21) $$\begin{array}{c} \frac{\int uW(r,u)du}{\int W(r,u)du}=\frac{1}{2\pi }\nabla \phi \left(r\right). \end{array}$$

Substituting Equation (21) into Equation (20) leads Equation (9), which is exactly Teague’s TIE. Therefore, Teague’s TIE is only a special case of GTIE when the optical field is completely temporally and spatially coherent. The GTIE, in contrast, explicitly accounts for effects due to spatial and temporal partial coherence, so it is valid for a much wider range of beams.

#### 5.2.2. Generalized Definition of “Phase” in Phase Space

Since the TIE applied to partially coherent fields reconstructs only a 2D function, it is insufficient to characterize the 4D coherence function [80]. However, one can still measure a through-focus intensity stack applying Teague’s TIE to reconstruct a 2D function in this plane, although the meaning of “phase” is clearly different from the one in a perfectly coherent field. As early as 1984, Streibl [119] used the 4D mutual intensity function to reformulate TIE in order to make it explicitly account for the partial coherence. He also pointed out that the phase retrieved by Teague’s TIE can still be related to the phase of the object (not the field), provided that the intensity distribution of the primary is axis-symmetric about the optical axis. Paganin and Nugent [81] have interpreted the “phase” of a partially coherent field as a scalar phase whose gradients become the normalized transverse time-averaged Poynting vector of the field. Based on the spectrum decomposition of a polychromatic field, Gureyev et al. [170] proposed an alternative interpretation of “phase” with the generalized Eikonal. Petruccelli et al. [172] interpreted the phase retrieved from the TIE as a scalar–valued function whose gradient is related to the transverse spectral density flow of the field.

The research work mentioned above quantitatively clarifies the meaning of phase and the applicability of TIE under partially coherent illuminations. However, the conventional space-time correlation quantities are involved to describe the properties of the partially coherent light, which makes the mathematical expression rather complicated, and the conclusions finally reached are difficult to interpret. In contrast, the phase-space GTIE allows us to create a more physically intuitive and generalized definition of “phase”. As shown in the left-hand side (LHS) of Equation (21), the generalized phase is *a scalar potential whose gradient yields the first order frequency moment of the WDF*, which is just the average spatial frequency at a particular location. In the optical context, we can intuitively interpret W(r,u) as the energy density of the ray passing through r with a frequency (direction) u. Thus, Equation (21) suggests that the time-averaged flux lines are defined as the orthogonal trajectories to the generalized phase, and they share the same direction of the average Poynting vector [81]. However, due to its possibility for negativeness, the WDF is not a rigorous energy density function [190,191]. However, there are no problems if the WDF is used to represent other measurable quantities, such as the intensity and the time-averaged Poynting vector in Equation (21), which are always non-negative.

#### 5.2.3. Light Field Moment Imaging Using Transport of Intensity Equation

We have already introduced LFMI, a computational light-field retrieval method, in Section 3. Now, we establish the close connection between the TIE and light ray field. Essentially, both of the two techniques look for complete descriptions of a field, but from different perspectives. Light ray field (radiance) represents all possible light rays as a function of position and direction, while, in coherent imaging, such information is encoded in the amplitude and phase of the 2D complex field. The 2D intensity and phase provide full knowledge about the complex field so that we can predict its behavior perfectly. For example, we can accurately propagate the beam to an arbitrary plane or computationally emulated different types of imaging modality, such as the Zernike phase contrast and DIC without using real optical hardware [78,164,192,193]. Obviously, representing a coherent complex field over 2D plane with the 4D phase-space expression is highly redundant. This redundancy leads to a highly localized WDF but is usually accompanied with oscillations due to the phase-space interference [187,194,195]. For a slowly varying object, the phase-space redundancy becomes more apparent since the phase-space oscillations disappear and the WDF occupies only a single slice in phase space [173]:

(22) $$\begin{array}{c} W\left(r,u\right)=I\left(r\right)\delta \left[u-\frac{1}{2\pi }\nabla \phi \left(r\right)\right]. \end{array}$$

**Figure 21:**
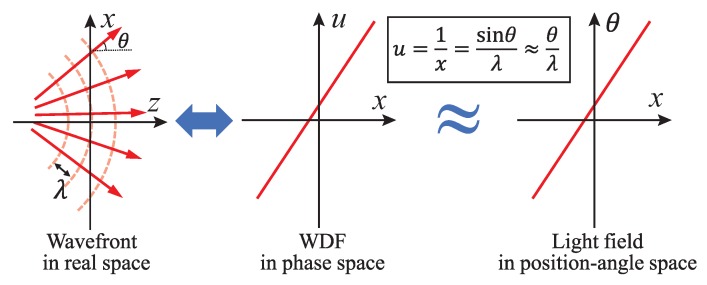
The WDF and light field of a smooth coherent wavefront. Phase is represented as the localized spatial frequency (instantaneous frequency) in the WDF representation. Rays travel perpendicularly to the wavefront (phase normal), (Reprinted from Optics and Lasers in Engineering, 71, Chao Zuo, Qian Chen, Lei Tian, Laura Waller, Anand Asundi, Transport of intensity phase retrieval and computational imaging for partially coherent fields: The phase space perspective, 13, Copyright (2015), with permission from Elsevier [173].)

By removing negative values, the form of the WDF given above is a true energy probability distribution, revealing the geometric ray or energy flow at a single position travels along a single direction described by the phase normal, as demonstrated in Figure 20a and Figure 21a. This allows phase measurement simplified by measuring the ray directions. One example is the Shack–Hartmann sensor [196]. Figure 21 visualizes a smooth coherent wavefront and its two phase-space representations (WDF and light field) with a simple correspondence of θ=λu.

**Figure 22:**
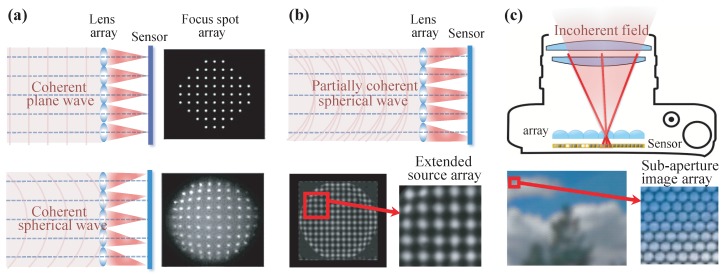
Principle of the Shack–Hartmann sensor and light field camera. (**a**) for coherent field, the Shack–Hartmann sensor forms a focus spot array sensor signal; (**b**) for a partially coherent field, the Shack–Hartmann sensor forms an extended source array sensor signa; (**c**) for incoherent imaging, the light field camera produces a 2D sub-aperture image array.

It is more complicated when the field is not strictly coherent. Generally, the phase-space WDF constitutes a rigorous and non-redundant representation for partially coherent fields. The 2D amplitude and (generalized) phase are insufficient to determine the field precisely [80,197]. From the perspective of geometric optics, for each point on the beam, there exist numerous light rays with different directions, and they fan out to create a 2D distribution, which accounts for the higher dimensionality of a partially coherent field, as illustrated in Figure 22b. The complete characterization of the 4D coherence function (so-called coherence measurement or coherence retrieval) has always been an active research area. The representative approaches include direct interferometric measurement [198,199], phase-space tomography [200,201], multi-plane coherence retrieval [202,203], and windowed Fourier transform approach [204,205]. All of these techniques require extra measurement dimension (by introducing an astigmatic lens) and much more intensity measurements. If the field exhibits significant spatial incoherence, the phase-space negativity and oscillations simply smooth out, and the WDF again approaches the radiance or the light field. In the computer graphics community, the light field camera, as a counterpart of the Shack–Hartmann sensor, also allows joint measurement of the spatial and directional distribution of the incoherent light field [29,31]. The 4D light field enables us to apply ray-tracing techniques to compute synthetic photographs, estimate depth, refocusing and viewing, perspectively [31,41]. However, it significantly sacrifices spatial resolution as compared to conventional imaging systems.

As Equation (21) shows, the generalized phase of the field is a scalar potential whose gradient is equal to the frequency moment of the WDF. Applying the correspondence between WDF and light field [68], i.e., L(x,θ)≈W(r,λu) to Equation (21), we can describe the phase gradient in terms of the light field:

(23) $$\begin{array}{c} \frac{\int \theta L(r,\theta )d\theta }{\int L(r,\theta )d\theta }=k^{-1}\nabla \phi \left(r\right). \end{array}$$

**Figure 23:**
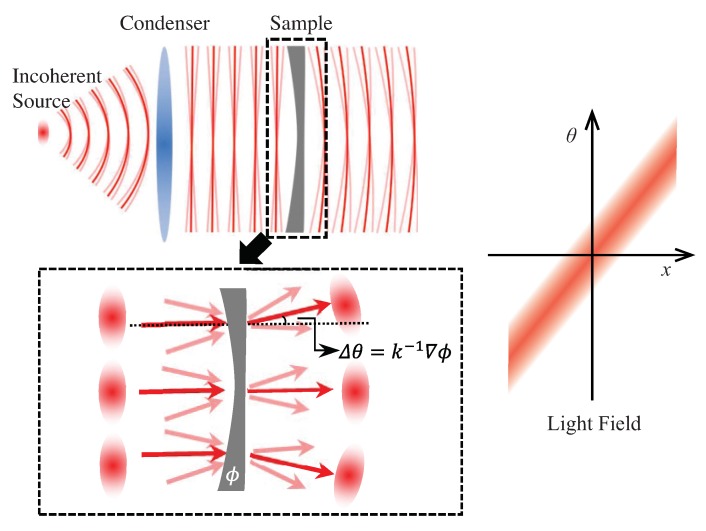
Light field representation of a slowly varying object under spatially stationary illumination. The sample exhibits angle-shift invariance: at each location, the direction of each incident ray shifts by the amount of object phase gradient; (Reprinted from Optics and Lasers in Engineering, 71, Chao Zuo, Qian Chen, Lei Tian, Laura Waller, Anand Asundi, Transport of intensity phase retrieval and computational imaging for partially coherent fields: The phase space perspective, 13, Copyright (2015), with permission from Elsevier [173]).

This equation shows that the phase gradient is related to the angular moment of the light field. Put simply, the angular moment is just the centroid of the light field, i.e., the average direction of light at one given position. Based on Equation (23), Zuo et al. [173] proposed and verified two important conclusions: (1) 4D light field contains 2D (generalized) phase information (phase can be recovered by analyzing the light field image): the phase gradient can be easily recovered from the 4D light field by spot centroid detection, which is similar to the Shack–Hartmann wavefront sensor [196]. However, for fields with different degrees of spatial coherence, the characteristics of the raw light field images are also completely different. For coherent wavefronts, geometric light ray at a single position travels only along a single direction, so the Shack–Hartmann sensor forms a focus spot signal array, as illustrated in Figure 22a. For partially coherent fields, geometric rays at a single position travel in various directions, forming a 2D extended source array instead, as shown in Figure 22b. For the field exhibits significant spatial incoherence, the rays at one given position travel to all possible directions, producing a sub-aperture image array in the image sensor, as illustrated in Figure 22c. (2) Though in general TIE cannot recover the complete 4D light field, the retrieved phase is related to the first angular moment of the light field. Furthermore, for some simplified conditions, (e.g., a slowly varying/non-scattering/spread-less specimen under spatially stationary illumination), the 4D light field is highly redundant (as shown in Figure 23). When the specimen is spreadless, it does not change the angular distribution of the incident field (no scattering), which is fully determined by the source distribution, and the direction of each incident ray is shifted as a function of the phase gradient of the object (refraction). This is why, in Figure 22b, the Shack–Hartmann sensor forms an extended source array sensor signal. Thus, with the knowledge of the source intensity distribution (angular distribution of the light field) and the phase of the object ϕ(r) retrieved from the TIE (angular moment of the light field), the 4D light field can be fully retrieved.

As we introduced in Section 3 [26], LFMI is an example of applying the idea of TIE to realize light ray field imaging. It has been proven that the partial differential equation used in LFMI (Equation (7)) is a variant of TIE at the geometric optics limit [123]. Therefore, any TIE solvers and axial derivative estimation approaches can be applied to LFMI. For example, in 2015, Liu et al. [67] adopted the high-order finite difference method to optimize the axial derivative estimation, improving the image quality and signal-to-noise ratio of LFMI. As we mentioned earlier, the phase retrieved by TIE can only be used to reconstruct the first angular moment of the 4D light field, which is exactly the physical meaning of “moment” in LFMI.

### 5.3. Hologram Synthesis from Focal Stack Measurements

The essential equivalence of light wavefront and ray field allows the interchangeable compatibility between them. Hologram synthesis from light ray field [66,206,207] gets benefits from this fact. This lets holographic imaging enable a breakthrough for the interferometric requirement, and makes a hologram of the real world more practical. On the other hand, the 2D hologram can be used to store a 4D light ray field, and makes a fast transfer of it possible, which is of great concern for the light ray field based glasses-free 3D display broadcast [12].

Except holographic stereogram [181] and synthesizing hologram from the light ray field [66,179,206,207], obtaining the hologram from depth measurements has also been proved possible. Not surprisingly, it has been proven that a Fourier hologram H(u,v) can be synthesized directly from depth measured photographic images with [53,54]

(24) $$\begin{array}{c} H\left(\rho \right)=\frac{\sum_{q=-Q}^{N}ℑ\left[I_{i}\left(r;q\Delta z_{i}\right)\right]\exp\left\{-j\frac{\pi q\Delta z_{i}}{\lambda f^{2}}\rho^{2}\right\}}{\sum_{q=-Q}^{Q}ℑ\left[h(r;q\Delta z_{i})\right]\cos\left\{\frac{\pi q\Delta z_{i}}{\lambda f^{2}}\rho^{2}\right\}}, \end{array}$$

where ρ is the coordinate of the Fourier spectrum plane, 2Q+1 is the number of depth measurements, and $\Delta z_{i}$ is the depth interval between two adjacent measurements. *ℑ* is the Fourier transform operator, λ is the wavelength used in hologram synthesizing, and *f* is the focal length of the virtual Fourier lens. Equation (24) has been proven to be equivalent to the Fourier hologram representation of a 3D object $\sum_{n=1}^{N}O\left(x,y,n\Delta z\right)$ [52], which can be reformed to

(25) $$\begin{array}{c} H\left(\rho \right)=\sum_{n=1}^{Q}\exp\left\{-j\frac{\pi n\Delta z}{\lambda f^{2}}\rho^{2}\right\}\int O\left(r;n\Delta z\right)\exp\left\{j\frac{2\pi }{\lambda f}r\rho \right\}dr. \end{array}$$

In this case, the measurements should cover the whole object space along the optical axis. As the issues in light ray reconstruction from depth measurements, the hologram synthesis shows the same problem, which needs further investigation. These kinds of issues also happen in the hologram synthesis from a light ray field [43,206].

## 6. Conclusions

In this paper, we reviewed 3D imaging techniques that are based on depth measurements in two aspects: ray-based and wavefront-based imaging techniques.

Depth measurement based light ray field imaging has been developed for only a few years. The related techniques can be divided into two types: LFBP and LFMI. Not being segmented by array-like devices, the spatial resolution of the reconstructed light-ray field is only limited by the camera NA. As these methods do not require any special equipment like lens array or coded masks, they are easy to be implemented. Despite their spatial resolution, the accuracy, noise, depth resolution and occlusion are the main issues. In general, LFBP can achieve a more accurate light field reconstruction while LFMI only reconstructs perspective-like images. The defocus information that acts as noise in LFPB needs additional image processing [51,63] or iterative approaches [71,72] to suppression. However, both image processing and iterative processes are not the best choice for achieving high depth resolution light field. The reason is that the depth resolution is directly related to the depth interval of the measurements. A high depth resolution thus can be achieved by taking a large amount of measurements with small depth intervals. This causes difficulty for noise suppression. To our knowledge, this has not been studied yet. In addition, taking a large amount of measurements is time-consuming or requires extra experimental setups [64]. The other issue that has not been well studied is the occlusion, which always exists in all of these techniques, even though it can be improved a little by iteration [71,72]. The summary of the comparison among the existing techniques is listed in Table 1. It is clear that there is a space-time compromise between the depth measurement-based and array-based light field imaging. Currently, none of them is perfect. More accurately, high depth resolution, and occlusion-free light field imaging from only a few depth measurements should be further studied. Per the description in Section 5, the connection between ray-based and wavefront-based light field imaging has been well analyzed: thinking light ray field from the viewpoint of wavefront may promote both of the research fields. One quotable example is the LFMI. Even though it cannot reconstruct an exact light field by now, it opens up a new concept of light field imaging.

The wavefront-based imaging has been proven to show a close connection to the ray-based 3D imaging. The phase gradient in wavefront based imaging is related to the first angular moment of the light ray field. Both LFMI and Shack–Hartmann sensor show this fact, which makes the wavefront-based imaging techniques have much in common with the depth measurement based light ray field imaging techniques.

Wavefront-based 3D imaging techniques are mainly focused on achieving the complex field of the wavefront. Both the iterative and quantitative approaches have been studied for several decades and are well-developed. The issues of the iterative phase retrieval techniques are compared in Table 2. Despite the limitations of each technique, the iterative phase retrieval remains a popular technique for wavefront reconstructions due to the fact that the optimal transfer function is object-dependent and due to the simplicity of its implementation. However, iterative phase retrieval based on scalar diffraction theory works under coherent illumination, which limits its application. TIE has been proved as a compensation to an iterative phase retrieval technique.

The development of TIE opens up a new epoch in phase retrieval and 3D imaging. i.e., from interferometric to non-interferometric, from iterative to deterministic, from phase measurement to light-field reconstruction, and from completely coherent to partially coherent or even incoherent. In this work, we reviewed the TIE’s basic principle, solutions, axial derivative estimation, and the generalization for partially coherent illuminations and light field imaging. Techniques about TIE solution, boundary conditions, phase discrepancy, axial derivative are compared in Table 3. Since the TIE is valid under partially coherent illuminations, it is possible to realize quantitative phase imaging based on a conventional microscope. Thanks to the built-in Köhler illumination as well as well-optimized optics inside a microscope, it is very easy to obtain high-quality phase images with diffraction-limited resolution and without any coherent noise. The single-beam optical configuration makes it very stable towards the vibration and other environmental disturbance. However, there are still several limitations in TIE that need to be addressed in the future. First, the phase measurement accuracy achieved is still much lower compared with interferometry. Second, TIE can only be applied under paraxial approximation and the intensity must be strictly positive. When there exist zero intensity point or phase vortex, it is very challenging to recover the phase distribution correctly based on TIE [208,209]. Finally, as we mentioned in Section 5.2, TIE can only recover the 2D light field moment instead of the full 4D light field. How to reconstruct a general high-resolution 4D light field by introducing additional information, e.g., low-resolution Shack–Hartmann sensor, combining with the through-focus intensity stack is a very interesting direction for future research.

Due to the difference of light sources and the imaging characteristics, ray and wavefront based imaging techniques have different applications. The wavefront-based 3D imaging, is more efficient for surveying micro details, and is thus well applied in microscopy applications [11]. The light rays are a more sparse sampling of the light field. Therefore, it is usually used for macro applications, such as displays [12]. However, the essential equivalence and close connection of the two have a mutual beneficial relationship, make them interchangeable, and share computational algorithms and hardware concepts. This mutual beneficial relationship also results in combination of them—for example, the light ray field can be properly combined with a wavefront technique for broader applications and better imaging performance [182]. In conclusion, even though there is no perfect technique for achieving good imaging quality (resolution, noise), time cost and system complexity at the same time, we believe that more sophisticated techniques will appear if we achieve mastery through a comprehensive study of the subjects. 

## Figures and Tables

**Table 1:** Comparison of light ray field reconstruction techniques.

	Accuracy	Noise	Occlusion	Time Cost
LFBP I ${}^{a}$	Moderate	High	Exists	Low
LFBP II ${}^{b}$	Moderate	Low	Exists partially *^e^*	High
iLFBP ${}^{c}$	High	Low	Exists less	High
LFMI ${}^{d}$	Low	High	Exists	Low

${}^{a}$ [62]; ${}^{b}$ [51,63]; ${}^{c}$ [71,77]; ${}^{d}$ [26]; *^e^* Occlusion exists in [51] and does not exist in [63].

**Table 2:** Comparison of iterative phase retrieval techniques.

	Techniques	Pros	Cons
Algorithm	GS	2 images	Error
			Stagnation
			Local minima
	GDS	Moderate fast	low
	HIO	Effective	
Contraints	Amplitude mask	Fewer iteration	Attenuation
	Phase mask	Fewer iteration	Fabrication
	Known pattern	Fewer iteration	
	Multi-depth	Resolution	Time cost
		Single position/shot possible	Possible experimental complexity
	Multi-wavelength	Resolution	Time cost
		Single position	Expensive
	Multi-angular	Resolution	Experimental complexity
	Structure illumination	Resolution	Experimental complexity
	Synthetic aperture	Resolution	Experimental complexity

GS: Gerchberg–Saxton; GDS: gradient decent search; IO:hybrid input-output.

**Table 3:** Comparison of TIE techniques.

Issues	Techniques	Pros	Cons
TIE solvers	Green’s function ${}^{a}$	Theoretical analysis	Computation-extensive, memory-demanding
	Multi-Grid ${}^{b}$	Simple and fast	Low-frequency noise,
	Zernike polynomials ${}^{c}$	Precisely represent the optical aberration	only for circular regions, difficult to follow details
	FFT ${}^{d}$	Fast, easy to implement, incorporate regularization in reconstruction	Imply periodic boundary conditions
	DCT *^e^*	Fast, inhomogeneous Neumann boundary condition	Rectangular aperture, required to limit FOV
	Iterative DCT ${}^{f}$	Inhomogeneous Neumann boundary condition, arbitrarily shaped apertures	Need several iterations
Boundary conditions	Homogeneous Dirichlet/Neumann ${}^{g}$	Easy to apply, can be implemented by different solvers	“Flat” boundary phase
	Periodic ${}^{h}$	Can be implemented by FFT-based solver	Periodic boundary phase
	Inhomogeneous Dirichlet ${}^{i}$	-	Boundary phase required
	Inhomogeneous Neumann ${}^{j}$	Can be measured by introducing a hard aperture	-
Phase discrepancy	Picard-type iteration ${}^{k}$	Can compensate the phase discrepancy	Need 2-4 iterations
Axial derivative	2-planes ${}^{l}$	Less intensity acquisition	Noise-resolution trade off
	Multi-planes ${}^{m}$	Higher resolution, better noise tolerance	More measurements

${}^{a}$ [20,124]; ${}^{b}$ [125,126]; ${}^{c}$ [127,128]; ${}^{d}$ [81,125,128,129]; *^e^* [130,131]; ${}^{f}$ [132]; ${}^{g}$ [133,134]; ${}^{h}$ [81,125,128,129]; ${}^{i}$ [20]; ${}^{j}$ [130,131,132]; ${}^{k}$ [135]; ${}^{l}$ [20]; ${}^{m}$ [136,137,138,139,140,141,142,143,144,145,146]; FFT: Fourier transform; DCT: discrete cosine transform; TIE: transport of intensity equation.
